# Plasma Rich in Growth Factors Promotes Autophagy in ARPE19 Cells in Response to Oxidative Stress Induced by Blue Light

**DOI:** 10.3390/biom11070954

**Published:** 2021-06-28

**Authors:** Carlota Suárez-Barrio, Susana del Olmo-Aguado, Eva García-Pérez, Luis Fernández-Vega-Cueto, Andrés Fernández-Vega Cueto, Begoña Baamonde-Arbaiza, Luis Fernández-Vega, Jesús Merayo-Lloves

**Affiliations:** 1 Instituto Universitario Fernández-Vega, Fundación de Investigación Oftalmológica & Universidad de Oviedo, 33012 Oviedo, Spain; carlotasb.8@gmail.com (C.S.-B.); evagp2002@gmail.com (E.G.-P.); lfvc@fernandez-vega.com (L.F.-V.-C.); afvc@fernandez-vega.com (A.F.-V.C.); bbaamonde@yahoo.es (B.B.-A.); prof.luis@fernandez-vega.com (L.F.-V.); merayo@fio.as (J.M.-L.); 2 Instituto de Investigación Sanitaria del Principado de Asturias, Avenida de Roma s/n, 33011 Oviedo, Spain

**Keywords:** PRGF, AMD, ARPE19, oxidative stress, autophagy, antioxidant, neuroprotection

## Abstract

Age-related macular degeneration (AMD) causes the degeneration of photoreceptors and retinal cells leading to vision loss in older subjects. Among possible exogenous risk factors, it has been recently proposed that long-term exposure to blue light could aggravate the course of AMD. In the search for therapeutic options, plasma rich in growth factors (PRGF) has been shown to enhance cell antioxidant pathways and protect photoreceptors against the harm produced by blue light, although its mechanism of action remains unknown. One possible mechanism, autophagy, is one of the most conservative cell renewal systems used in eukaryotes to destroy cellular components that have been damaged by some kind of insult. The oxidative stress of exposure to blue light is known to induce cell autophagy. In this study, we examined the combined effects on autophagy of blue light and PRGF in a retinal cell line, ARPE19. In response to treatment with both PRGF and blue light, we detected the modulated expression of autophagy markers such as NF-kB, p62/sqstm1, Atg5, LC3 and Beclin1, and inflammatory markers such as IL1B and IL18. Our findings suggest that PRGF promotes cell autophagy in response to exposure to blue light.

## 1. Introduction

Age-related macular degeneration (AMD) is one of the leading causes of blindness in elderly subjects [1,2,3]. This disease is the consequence of the degeneration of photoreceptors, which are specialized retinal cells with high energy requirements that convert light into electrical signals that are processed in the brain. Because of their high mitochondrial activity, photoreceptor cells generate large amounts of reactive oxygen species (ROS). To offset the oxidative stress produced by ROS, different antioxidant systems exist in the retina. However, several factors can lead to an overproduction of ROS, and this can disrupt many antioxidant pathways and finally lead to photoreceptor cell death [4,5,6,7,8,9,10,11,12]. One such exogenous factor is light. 

Blue light (400–500 nm) is the fraction of the visible spectrum that can be harmful to retinal cells [13,14,15,16,17,18,19,20,21,22,23,24,25,26]. That short wavelength light is absorbed by flavin and mitochondrial cytochrome constituents, causing mitochondrial membrane depolarization, a reduction in ATP synthesis and an increase in ROS production [15]. According to several of our studies examining the effects of blue light on retinal cells [27,28,29], this insult enhances ROS production and impairs the functionality of photoreceptors [30]. Our group has also shown that plasma rich in growth factors (PRGF) is able to reduce these impacts of blue light by stimulating antioxidant pathways, thus protecting cells against this damage. PRGF induces nuclear translocation of nuclear factor erythroid 2-related factor (Nrf2) stimulating heme-oxiganse-1 (HO-1) or glutamate-cysteine ligase (GCL) [28]. 

As this plasma is extracted from the patient’s own blood, an adverse immunologic response is avoided. The benefits of PRGF have been described in several medical fields such as odontology and traumatology [31,32,33,34,35,36,37,38,39]. In ophthalmology, PRGF has been used to treat corneal defects or dry eye [40,41,42,43,44,45,46,47,48,49]. 

Autophagy consists of transport via different systems of cytoplasmic components into the lysosome (vacuoles) and is among the most conserved processes of cell renewal found in eukaryotes. Based on structural and mechanistic features, the autophagy pathways found are classified into three types: macroautophagy (here referred to as autophagy), microautophagy and chaperon-mediated autophagy [50]. Autophagy is a catabolic process that activates the degradation of cellular components that are damaged via lysosomes through the formation of autophagosomes [51,52,53,54]. This mechanism is activated after cell exposure to different kinds of insult, such as oxidative stress or inflammation, and is thus a useful tool to protect cells [55,56,57,58]. 

**Figure 1:**
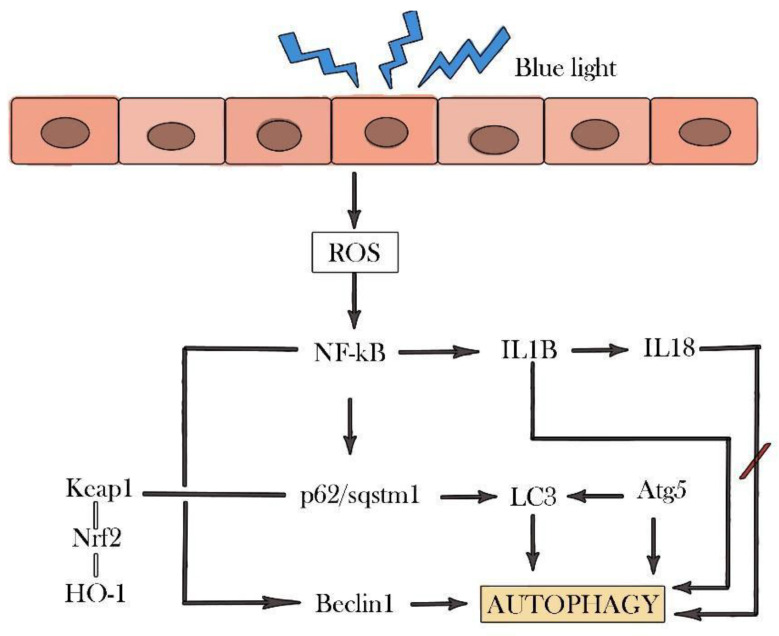
Autophagy activation pathways. Blue light increases ROS production in RPE cells, activating NF-kB, which modulates autophagy, antioxidant, and inflammatory pathways.

Besides inducing oxidative stress, blue light can also act as a pro-inflammatory agent. Hence to mitigate its harmful effects, blue light could induce the expression of markers that initiate antioxidant and anti-inflammatory pathways such as nuclear factor-kappa-light-chain-enhancer of activated B cells (NF-kB). NF-kB is a transcriptional factor whose expression is triggered in the presence of ROS, and this is followed by activation of both the proinflammatory and autophagy pathways (see Figure 1) [59]. The autophagy pathway is preceded by activation of sequestosome 1 (p62/sqstm1) [60], which promotes the turnover of poly-ubiquitin-proteins to the proteasome, regulating the activation of antioxidant pathways by binding to Kelch-like ECH-associated protein 1 (Keap-1) and modulating the release of Nrf2 from the cytoplasm to the nucleus. Here, Nrf2 activates the expression of other antioxidant molecules such as HO-1 [61,62,63,64,65,66,67,68], and also interacts with the autophagy marker microtubule-associated proteins light chain 3 (LC3) [53,57,69,70]. There are also different proteins, known as autophagy-related proteins (Atg), which control the whole process of autophagy activation by binding to each other and to other molecules to activate phagophore formation. For instance, expression of the cytosolic form of LC3, LC3I, is stimulated by Atg4 and Atg7. This is followed by binding of LC3I to phosphatidylethanolamine (PE) induced by Atg3, transforming it into the lipid form, LC3II. Next, LC3II is activated by Atg5-Atg12-Atg16 to bind to the phagophore for its elongation and remains bound until the autophagosome is linked to the lysosome. The LC3II portion that remains on the cytoplasmic side of the autophagosome membrane is delipided by Atg4 and recycled, while LC3II, located on the inner side, will be degraded with the phagolysosome after its fusion. Many researchers consider LC3 a good marker of autophagy as it is present from the initial stages of phagophore formation. A different autophagy pathway also exists that is independent of LC3 activation, carried out by Atg5-Atg7 [50,56,58,61,66,70,71,72,73,74,75,76,77,78,79]. The expression of NF-kB also reflects cleavage of B-cell lymphoma 2 (Bcl-2), an autophagy inhibitor protein, from Beclin1 [55,56,69,80,81,82], which is also a marker of autophagosome formation. 

NF-kB also modulates the proinflammatory pathway. When NF-kB is activated, it stimulates the formation of inflammasome through the expression of cytokines such as interleukin 1 beta (IL1B) and interleukin 18 (IL18). However, the combined actions of NF-kB and p62/sqstm1 also downregulate inflammasome expression, controlling both its activation and inhibition. While IL1B expression is induced during autophagy, IL18 expression is stimulated when autophagy is inhibited [55,68,83,84,85,86]. 

As exposure to short-wavelength light is a risk factor for eye diseases such as AMD, this study examines the role of autophagy induced by blue light as a therapeutic target for this disease. Our working hypothesis was that, as an antioxidant, PRGF could attenuate the damage caused by blue light in a human retinal cell line.

## 2. Materials and Methods

### 2.1. PRGF

Blood was obtained from 4 healthy donors (all women, mean age 33 ± 7 years) in accordance with principles of the Helsinki Declaration of 2013. Samples were placed in 9 mL tubes with 3.8% sodium citrate (Vacuette tube, Greiner Bio-One, Kremsmünster, Austria) and then centrifuged at room temperature (Endoret System, BTI Biotechnology Institute, S.L., Vitoria, Spain). After centrifugation, whole plasma was collected avoiding the leukocyte layer and transferred to a 15 mL tube. The plasma was mixed with calcium chloride for fibrinogen activation and incubated for 30 min at 37 °C, or until clotting. The supernatant was collected and heated (56 °C) for 1 h to inactivate the complement system. Next, the plasma was filtered, aliquoted, and kept at −20 °C until use (in less than 6 months).

### 2.2. Cell Culture Experiments

Human ARPE19 cells, retinal pigment epithelial (RPE) cell line (ATCC, Wesel, Germany), were grown in a culture medium consisting of DMEM-F12 solution (Sigma-Aldrich, St Louis, MO, USA) supplemented with 2% antibiotic penicillin/streptomycin (Sigma-Aldrich, St Louis, MO, USA) and 10% foetal bovine serum (FBS), and kept in a humidified atmosphere of 5% CO_2_ at 37 °C. The doubling growth time was approximately 60 h. Aliquots (100 μL or 2 mL) of the cell culture (approximately 10 × 10^4^ cells/mL) were taken and placed in 96-well plates or T75 flasks, respectively. After allowing the cells to settle (approximately 24 h for the 96-well plates and 72 h for the T75 flasks) the samples were subjected to the treatments indicated in Table 1. To deliver light to the cultures we used blue light LEDs (Electro DH SL, Barcelona, Spain) (465–475 nm, 400 lux, 18 W/m^2^). Time of light exposure was determined in previous experiments [28], where the lowest light intensity was selected in order to cause around 20% of viability reduction. Temperature was kept at 37 °C. The cells were subjected to one-hour of pre-treatment in the dark to allow them to settle in the new culture medium. 

### 2.3. Western Blotting

After the different treatments, ARPE19 cells were collected by scraping from the T75 flasks followed by centrifugation and resuspension in a cocktail lysis buffer that contained phosphatase and protease inhibitors (Sigma-Aldrich, Saint Louis, MO, USA). After freezing, thawing and sonication, the supernatant was collected with its protein content. Equal amounts of proteins were fractionated by electrophoresis using 12.5% polyacrylamide gels containing 0.1% SDS. Proteins were transferred to 0.22 μm nitrocellulose membranes and were incubated overnight at 4 °C with one of a set of primary antibodies (see Table 2). Proteins were detected using appropriate biotinylated secondary antibodies. The final nitrocellulose blots were developed with a 0.016% *w*/*v* solution of 3-amino-9-ethylcarbazole in 50 mM sodium acetate (pH 5.0) containing 0.05% (*v*/*v*) Tween-20 and 0.03% (*v*/*v*) H_2_O_2_. The colorimetric reaction was stopped with 0.05% sodium azide/PBST solution, and the density of the individual bands quantified using IMAGEJ software (U.S. National Institutes of Health, Bethesda, MD, USA).

### 2.4. RNA Extraction and mRNA Analysis

Total RNA was extracted from ARPE19 cells using the Illustra RNAspin Mini kit (GE Healthcare, Chicago, IL, USA). The purity of the RNA was then checked through the A260/A280 and A260/A230 ratio. Next, 0.5 μg of total RNA was used for linear conversion of RNA to cDNA using the High-Capacity RNA-to-cDNA Master Mix (Applied Biosystems, Waltham, MA, USA) following the manufacturer’s instructions (60 min at 37 °C, 5 min at 95 °C, and holding at 4 °C). Primers (see Table 3) were customised using PrimerBLAST and synthesized by Sigma-Aldrich (Sigma-Aldrich, St Louis, MO, USA). Gene expression was quantified by relative quantification in a 7500 Real-Time PCR System (Applied Biosystems, Waltham, MA, USA) using a Power SYBR Green PCR Master Mix (Applied Biosystems, Waltham, MA, USA) and the ∆∆Ct method. Each sample was analysed in triplicate for each of the experiments (*n* = 4). Data were analysed using SDS 1.4 software (Applied Biosystems, Waltham, MA, USA).

### 2.5. Statistical Analysis

All statistical tests were performed using the package GraphPad Prism version 7.0a for Mac (GraphPad Software, La Jolla, CA, USA). Data were compared between groups by one-way ANOVA. To compare mean differences among treatments, we used Tukey’s multiple comparison test and the Kruskal–Wallis multiple comparison test. Significance was set at *p* < 0.05.

## 3. Results

### 3.1. NF-kB

**Figure 2:**
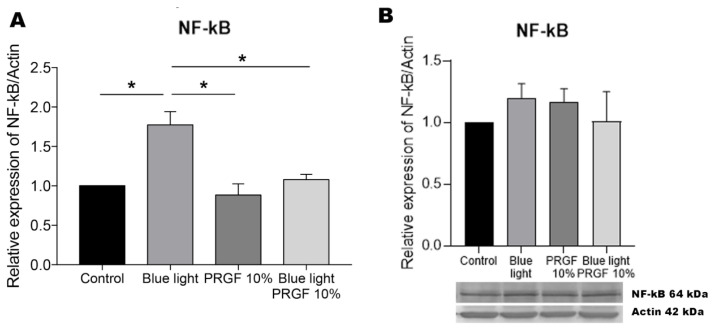
NF-kB gene expression and protein expression relative to the expression of actin. (**A**) NF-kB gene expression measured by qPCR. Results indicate that in response to blue light its gene expression was significantly increased. PRGF plus blue light treatment produced a significantly different effect to blue light alone, suggesting that PRGF was able to reduce the impacts of ROS. One-way ANOVA, Tukey’s multiple comparisons test, * *p* < 0.05 (*n* = 4). (**B**) NF-kB protein expression measured by Western blotting. Results indicate no significant differences in protein expression among the treatments. One-way ANOVA, Kruskal–Wallis multiple comparisons test (*n* = 4).

Our gene and protein expression results for NF-kB are illustrated in Figure 2. Exposure of the retinal cells to blue light led to the increased gene expression of this marker, which was significantly reduced in the presence of PRGF. Western blots revealed no significant difference in protein expression among the four different treatments. These findings could indicate the translocation of NF-kB to the nucleus to activate the different protective autophagy pathways. 

**Figure 3:**
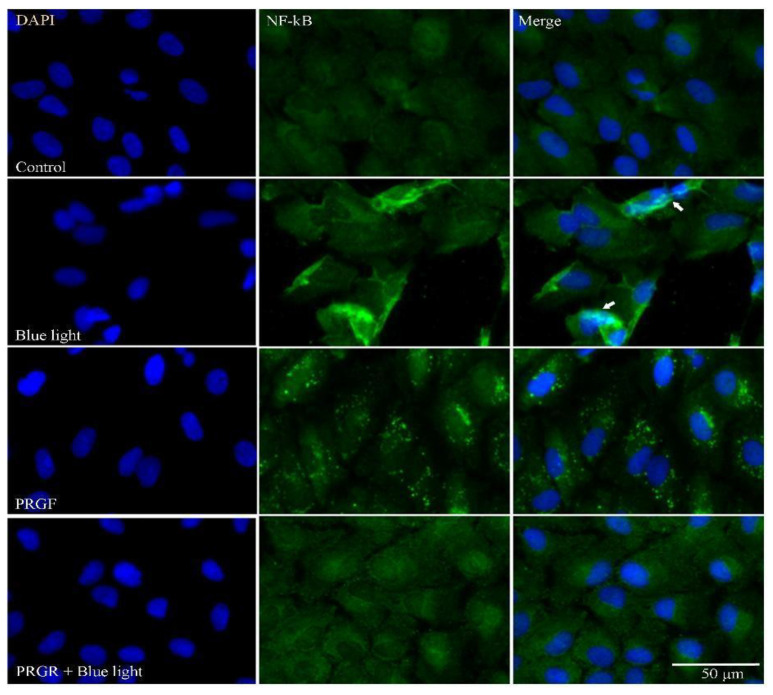
Immunofluorescence staining of NF-kB (green) and nucleus (DAPI, blue). Results indicate the increased presence of NF-kB in the cell nucleus in response to blue light. Treatment with PRGF alone led to a dotted pattern of NF-kB around the nucleus. White arrows point to NF-kB in the nucleus. Scale bar 50 µm (*n* = 4).

The translocation of NF-kB to the nucleus was confirmed by immunofluorescence staining. The images in Figure 3 show that in response to blue light treatment there is co-location of DAPI (nucleus stained blue) and NF-kB, indicating the localization of the marker in the nucleus after activation. We also observed that the PRGF treatment gave rise to a punctate pattern of staining for the marker in the perinuclear zone. This could suggest that PRGF induces the deployment of the marker around the nucleus in preparation for its actions if needed. This possibility needs to be addressed in future work. 

### 3.2. p62/sqstm1

**Figure 4:**
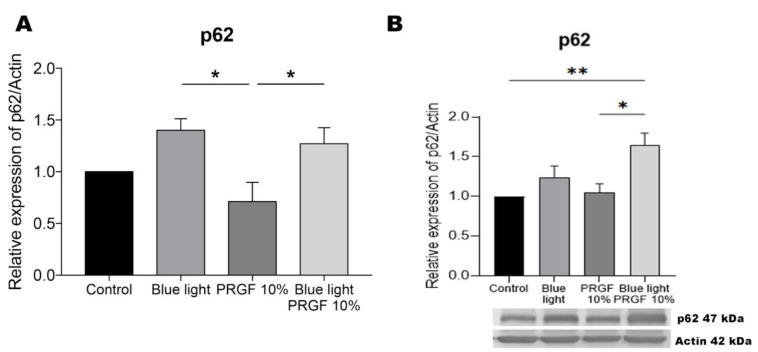
p62/sqstm1 gene expression, and protein expression relative to the expression of actin. (**A**) p62/sqstm1 gene expression measured by qPCR. Results indicate that in response to blue light alone, or in combination with PRGF, its gene expression was significantly increased compared to PRGF alone. One-way ANOVA, Tukey’s multiple comparisons test, * *p* < 0.05 (*n* = 4). (**B**) p62/sqstm1 protein expression measured by Western blotting. Results indicate that PRGF plus blue light led to a significant increase in the expression of this marker compared to the control and PRGF treatments. One-way ANOVA, Tukey’s multiple comparisons test, * *p* < 0.05, ** *p* < 0.005 (*n* = 4).

Our p62/sqstm1 gene expression results (Figure 4) indicate that blue light alone led to the increased expression of this marker compared to treatment with PRGF alone. In addition, when blue light was combined with PRGF, its expression was also significantly increased compared to the PRGF treatment alone. Our protein expression results for p62/sqstm1 confirmed that the treatment PRGF plus blue light caused its increased expression compared to the control and PRGF-alone treatments. Further, blue light treatment led to the increased, although not significant, expression of this marker.

### 3.3. Atg5

**Figure 5:**
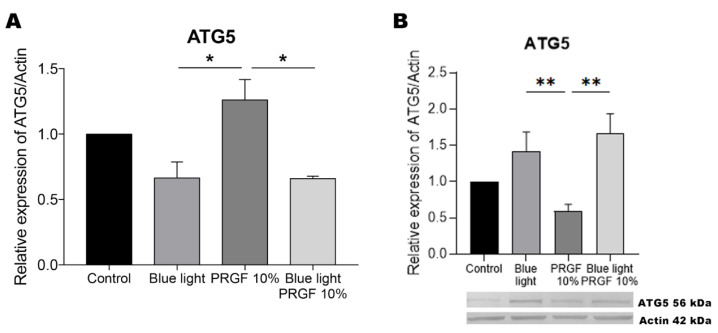
Atg5 gene expression, and protein expression relative to the expression of actin. (**A**) Atg5 gene expression measured by qPCR. Results indicate that in the presence of PRGF, its gene expression was significantly increased compared to the blue light treatment, combined or not with PRGF. One-way ANOVA, Tukey’s multiple comparisons test, * *p* < 0.05 (*n* = 4). (**B**) Atg5 protein expression measured by Western blotting. Results indicate that blue light, alone or combined with PRGF, led to a significant increase in the expression of this marker compared to the PRGF treatment. One-way ANOVA, Tukey’s multiple comparisons test, ** *p* < 0.005 (*n* = 4).

The next marker of autophagy examined, Atg5, showed enhanced expression in the presence of PRGF (Figure 5). This protein acts in the initial stages of autophagosome formation, suggesting its expression will be easier to study in the initial stages of exposure to blue light and/or PRGF treatment. Our Western blots, however, revealed the increased production of this marker in response to blue light, both alone and in combination with PRGF. This could suggest that the presence of this marker is induced in the initial stages of autophagy through its gene expression but that the protein remains activated until the final stages.

### 3.4. LC3

**Figure 6:**
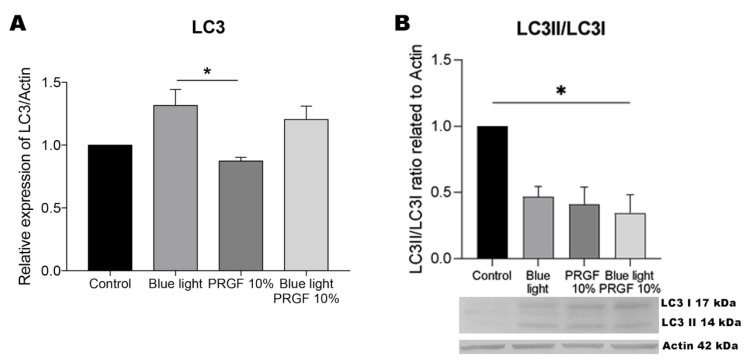
LC3 gene expression, and protein expression relative to the expression of actin. (**A**) LC3 gene expression measured by qPCR. Results indicate that in response to blue light, its gene expression was significantly increased compared to the PRGF treatment. It was also possible to see a difference between control and blue light treatments, however it was not significant (*p* = 0.1065). One-way ANOVA, Tukey’s multiple comparisons test, * *p* < 0.05 (*n* = 4). (**B**) LC3II:LC3I ratio of protein expression measured by Western blotting. Results indicate that PRGF plus blue light led to a significant increase in the expression of LC3I compared to the control treatment. One-way ANOVA, Tukey’s multiple comparison test, * *p* < 0.05 (*n* = 4).

The gene expression of LC3 was found significantly enhanced in the presence of blue light compared to the control and PRGF treatments (Figure 6). When blue light was combined with PRGF, the expression of this marker was also higher, but not significantly. In our protein expression experiments, we examined both the “inactivated” form (LC3I) and activated form (LC3II) of LC3 as the former needs to bind to PE to be activated and join to the phagophore for its elongation. The ratio LC3II to LC3I was decreased compared to control results indicating higher levels of LC3I than LC3II.

### 3.5. Beclin1

**Figure 7:**
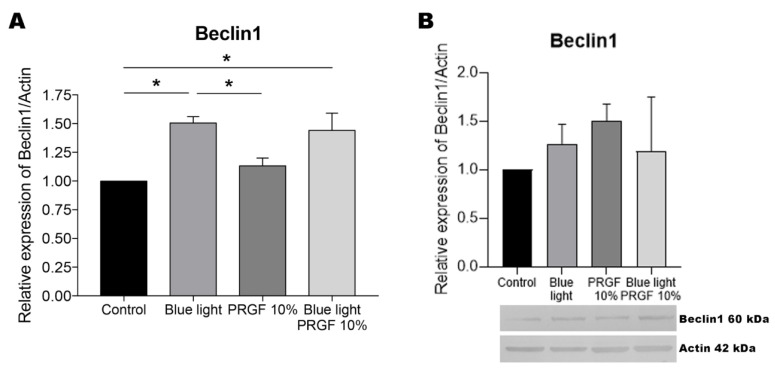
Beclin1 gene expression, and protein expression relative to the expression of actin. (**A**) Beclin1 gene expression measured by qPCR. Results indicate that in response to blue light with or without PRGF its gene expression was significantly increased compared to the control treatment. One-way ANOVA, Tukey’s multiple comparison test, * *p* < 0.05 (*n* = 4). (**B**) Beclin1 protein expression measured by Western blotting. Results indicate no significant difference in protein expression among the treatments. One-way ANOVA, Kruskal–Wallis multiple comparison test (*n* = 4).

The gene expression of Beclin1 was increased in response to blue light in combination or not with PRGF (Figure 7). Its protein expression was higher in response to treatment with PRGF alone, although the difference was not significant. No conclusions are available for the PRGF plus blue light treatment, as the resolution was not clear.

### 3.6. IL1B

**Figure 8:**
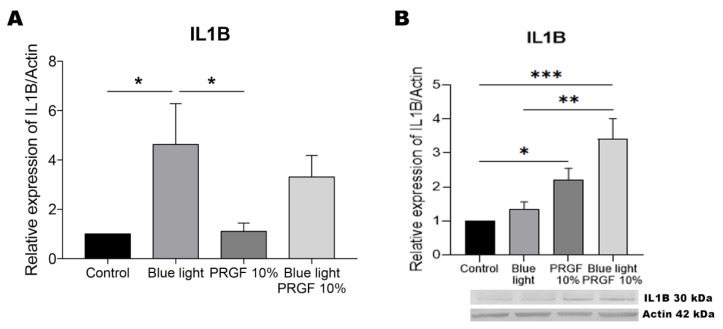
IL1B gene expression, and protein expression relative to the expression of actin. (**A**) IL1B gene expression measured by qPCR. Results indicate that in response to blue light this expression was significantly increased compared to the remaining treatments. One-way ANOVA, Tukey’s multiple comparison test, * *p* < 0.05 (*n* = 4). (**B**) IL1B protein expression measured by Western blotting. Results indicate that blue light plus PRGF led to a significant increase in the expression of this marker compared to blue light alone. In addition, PRGF alone also gave rise to the increased expression of this marker compared to control results. One-way ANOVA, Tukey’s multiple comparison test, * *p* < 0.05, ** *p* < 0.005 and, *** *p* < 0.0005 (*n* = 4).

Exposure of the retinal cells to blue light led to a significantly high increase in the gene expression of IL1B, while its protein expression was increased in response to PRGF when given alone or in combination with blue light (Figure 8).

### 3.7. IL18

**Figure 9:**
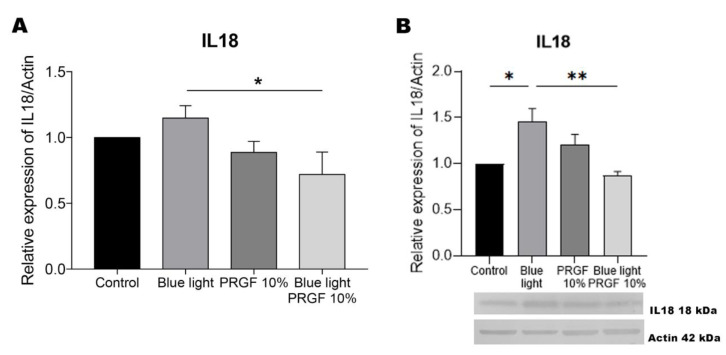
IL18 gene expression, and protein expression relative to the expression of actin. (**A**) IL18 gene expression measured by qPCR. Results indicate that in response to blue light alone the expression of the marker was increased compared to its combination with PRGF. One-way ANOVA, Tukey’s multiple comparison test, * *p* < 0.05 (*n* = 4). (**B**) IL18 protein expression measured by Western blotting. Results indicate that blue light led to a significant increase in the expression of this marker compared to the blue light plus PRGF and control treatments. One-way ANOVA, Tukey’s multiple comparison test, * *p* < 0.05 and ** *p* < 0.005 (*n* = 4).

Our results indicate that the gene expression of IL18 was enhanced by blue light compared to blue light plus PRGF, while its protein expression was significantly enhanced by blue light yet reduced by blue light plus PRGF (Figure 9).

## 4. Discussion

Autophagy is among the most regulated and conserved processes of cell renewal known [51,54]. It is regulated by the activation and inactivation of several markers in response to damage produced in some cell components through factors such as oxidative stress or inflammation. It has been well established that blue light increases the presence of ROS with possible harmful effects produced via the disruption of several retinal molecules. Although retinal cells have several antioxidant pathways for their protection, these might not be sufficient when ROS levels are elevated and/or maintained over long periods [5,56,83,87]. Further, blue light disrupts the activity of photoreceptors by dysregulating several proteins that ensure correct visual function. In work from our laboratory, PRGF was found to diminish the cell damage produced by blue light, as it is able to upregulate protective antioxidant pathways and also avoids the disruption of these proteins [21,22,75,88,89,90,91,92]. In the present study, we also noted that blue light is able to enhance autophagy and that this process is promoted by PRGF. 

NF-kB is a transcriptional factor that is activated following the production of ROS [66,93] regulating several systems including antioxidant and inflammatory pathways. The site of NF-kB activation is the cytoplasm, and this is followed by the activation of IL1B and the inflammasome [56,80,84]. NF-kB modulates its own inflammatory activity via the renewal of p62/sqstm1 in damaged mitochondria [66,68]. This acts as a negative feedback loop, controlling the activation of inflammation but also preventing tissue damage. Our results indicate that NF-kB gene expression is significantly exacerbated in response to blue light. We propose that the blue light insult increases the presence of ROS, and that NF-kB is activated to reduce the harm produced. However, when blue light was combined with PRGF, the expression of this marker was reduced, although it did not reach basal levels. Remarkably, on its own, PRGF was found to reduce the expression of this marker compared to the control treatment. This suggests that PRGF attenuates ROS and therefore the expression of NF-kB. We were unable to detect differences in the protein expression of this marker among the treatments. This could be because NF-kB is translocated to the nucleus to activate the expression of other protective pathways and thus it cannot be detected in the cytoplasm.

The protein p62/sqstm1 acts as an adapter between the ubiquitin-proteasome system (UPS) and the autophagy-lysosome pathway (ALP) [66]. When the proteasome is overwhelmed, p62/sqstm1 controls ALP, as it has an LC3 interacting region. This protein also features an antioxidant response element (ARE) which is regulated by the Keap1-Nrf2 pathway. In the presence of ROS, p62/sqstm1 binds to Keap1, releasing Nrf2 from the cytoplasm, which travels to the nucleus to activate the expression of other antioxidant molecules such as HO-1 [57,61,65,69]. Our p62/sqstm1 gene expression analysis revealed that blue light led to a slight increase in its expression. However, the presence of PRGF in combination with blue light significantly raised this level of expression. This could mean the activation of p62/sqstm1 expression in an effort to enhance antioxidant and autophagy pathways. Our Western blots results confirmed that treatment with PRGF and blue light highly increased the protein expression of this maker. In prior work [28], we showed that PRGF promoted antioxidant pathways when blue light was present. This is consistent with our finding of the increased expression of p62/sqstm1 induced by PRGF in response to blue light, suggesting promotion of the protective pathways it oversees.

It is clear that autophagy is a tightly controlled process. Several authors have reported the presence of LC3 as a sign of autophagy. LC3 is expressed in its “inactive” form, LC3I, which undergoes lipidation by binding to PE and is transformed into LC3II. LC3II binds to the phagophore membrane and, via other regulators, elongates the phagophore until it becomes an autophagosome [50,53,70,75,94,95]. The proteins that control the autophagy process are called autophagy-related proteins (Atg). Accordingly, Atg5 forms a complex with Atg12 [79,96]. When these proteins detect LC3II, they bind to Atg16 and help LC3II to anchor to the phagophore membrane, where it carries out its maturation function [86]. Our Atg5 gene expression analysis revealed that PRGF led to a great increase in its expression, showing an opposite pattern compared to its protein expression. This protein acts in the initial stages of autophagosome formation, so this finding could suggest its easier detection at the start of this process. Accordingly, our Western blot assay revealed that blue light, alone or in combination with PRGF, increased the protein expression of this marker. These results therefore support this notion that Atg5 expression is induced in the initial stages of cell damage, as it helps LC3II binding to the phagophore for its elongation, but the protein remains activated for a longer period. However, there is evidence to suggest that the expression of Atg5/Atg12 is controlled by circadian rhythm such that it could follow a cycle [75,97,98,99,100]. LC3 gene expression is increased in response to blue light and slightly increased when blue light is combined with PRGF. This suggests that blue light enhances autophagy, whose objective is to destroy and recycle all damaged cellular fractions. Several studies have shown that LC3 expression is greatly elevated in the initial stages of autophagy owing to its role in autophagosome maturation. Nevertheless, exposure to blue light was found here to induce the expression of this marker during the whole experiment. Results regarding the expression of this protein could be misleading. In order to detect the real amount of protein that is carrying out its function, it is important to consider both LC3I and LC3II. Hence, when retinal cells were treated with blue light plus PRGF, LC3I expression was higher than that of LC3II. This could indicate greater protein expression levels in early stages of autophagy, and once the autophagosome is formed and mature, LC3I does not require conversion into LC3II. Moreover, it might not be necessary to promote the expression of the gene when the protein is not being activated. Song et al. observed that the protein expression of LC3 follows an opposite pattern to that of p62/sqstm1, such that p62/sqstm1 expression was higher when a lower amount of LC3II was detected [66]. 

NF-kB also activates the release of Beclin1 from Bcl-2, an autophagy inhibitor. Like LC3, Beclin1 plays a role in phagophore nucleation and autophagosome elongation [81]. Our gene expression results revealed that blue light increased its expression but also when it was combined with PRGF. In Western blots we detected that PRGF alone stimulates its protein expression, although results were not significantly different. Despite our unclear results for the treatment blue light plus PRGF, these suggest higher expression levels of this marker than control levels, and therefore that autophagy might be stimulated.

As mentioned earlier, NF-kB also plays an important role in regulating inflammation. Further, NF-kB modulates its own pro-inflammatory function acting through negative feedback, controlling inflammasome formation and therefore preventing tissue damage. Several studies have linked different cytokines with the regulation of autophagy. When NF-kB is activated after the detection of ROS, cytokines such as IL1B and IL18 are expressed [55,62,84,101,102,103,104]. In effect, it has been widely described that IL1B expression is stimulated in the event of autophagy. Our qPCR results indicate the intensely increased gene expression of this marker in response to blue light. In addition, as IL1B expression is modulated in the presence of ROS, we observed that treatment with both PRGF and blue light resulted in the reduced expression of IL1B. However, our Western blots revealed an increase in the expression of this marker when blue light was combined with PRGF. We propose this finding is related to the role of this cytokine in the activation of autophagy. While IL18 is usually expressed when autophagy is inhibited, our data indicate that treatment with PRGF reduced its gene and protein expression, suggesting that autophagy was not inhibited.

## 5. Conclusions

Our results confirm that autophagy is an intricate process that is regulated in very different ways. Despite this, we were able to see in our retinal cell culture model that, because of the damage it causes to cell structures, blue light enhances autophagy, but when combined with PRGF it stimulates this system even further. PRGF alone did not impair the different cellular mechanisms, but it was able to prepare the cell machinery to respond to this insult. 

## Figures and Tables

**Table 1:** Treatments.

Treatments	Culture Medium	Dark/Blue Light
Control	DMEM F12 + FBS 1%	Dark 19 h
Blue light	DMEM F12 + FBS 1%	Dark 1 h + blue light 18 h
PRGF	PRGF 10% + DMEM F12 + FBS 1%	Dark 19 h
Blue light + PRGF	PRGF 10% + DMEM F12 + FBS 1%	Dark 1 h + blue light 18 h

**Table 2:** Antibodies used for the Western blots.

Antibody	Reference (RRID)	Species	Dilution	Company
**Primary Antibodies**				
Actin	Millipore Cat# MAB1501, RRID:AB_2223041	Mouse	1:4000	Millipore, Burlington, MA, USA
NF-kB	Santa Cruz Biotechnology Cat# sc-109, RRID:AB_632039	Rabbit	1:100	Santa Cruz, Dallas, TX, USA
p62/sqstm1	Abcam Cat# ab56416, RRID:AB_945626	Mouse	1:500	Abcam, Cambridge, UK
ATG5	Santa Cruz Biotechnology Cat# sc-133158, RRID:AB_2243288	Mouse	1:500	Santa Cruz, Dallas, TX, USA
LC3	Abcam Cat# ab192890, RRID:AB_2827794	Rabbit	1:2000	Abcam, Cambridge, UK
Beclin1	Santa Cruz Biotechnology Cat# sc-48341, RRID:AB_626745	Mouse	1:1500	Santa Cruz, Dallas, TX, USA
IL1B	Abcam Cat# ab9722, RRID:AB_308765	Rabbit	1:100	Abcam, Cambridge, UK
IL18	Abcam Cat# ab191152, RRID:AB_2737346	Rabbit	1:250	Abcam, Cambridge, UK
**Secondary Antibodies**				
Anti-mouse	Vector Laboratories Cat# BA-9200, RRID:AB_2336171	Goat	1:300	Vector labs, Burlingame, CA, USA
Anti-rabbit	Vector Laboratories Cat# BA-1000, RRID:AB_2313606	Goat	1:300	Vector labs, Burlingame, CA, USA

**Table 3:** Primers used for qPCR.

Gene	ID	Forward	Reverse
*Actin*	NM_001101.4	5′-ATTCCAAATATGAGATGCGTTGTT-3′	5′-GTGGACTTGGGAGAGGACTG-3′
*NF-kB*	NM_001165412.2	5′-CAGATGGCCCATACCTTCAAAT-3′	5′-CGGAAACGAAATCCTCTCTGTT-3′
*p62*/*sqstm1*	NM_001142298.2	5′-TGTGAATTTCCTGAAGAACG-3′	5′-TCGATATCAACTTCAATGCC-3′
*ATG5*	NM_001286106.2	5′-CCCTCTTGGGGTACATGTCT-3′	5′-CGTCCAAACCACACATCTCG-3′
*LC3*	NM_032514.4	5′-GTTGGTCAAGATCATCCG-3	5′-TTTCTCCTGCTCGTAGATG-3
*Beclin1*	NM_001313998.2	5′-CAGTATCAGAGAGAATACAGTG-3′	5′-TGGAAGGTTGCATTAAAGAC-3′
*IL1B*	NM_000576.3	5′-GGCTGCTCTGGGATTCTCTT-3′	5′-ATTTCACTGGCGAGCTCAGG-3′
*IL18*	NM_001243211.2	5′-TGCAGTCTACACAGCTTCGG-3′	5′-GTTTGTTGCGAGAGGAAGCG-3′

## Data Availability

All the obtained data used to support the findings of this study are available from the corresponding author upon reasonable request.
